# A Typical Presentation of Cesarean Section Scar Endometriosis: A Case Report

**DOI:** 10.7759/cureus.49884

**Published:** 2023-12-03

**Authors:** Hamdi Al Shenawi, Noor Al Shenawi, Noor A Al Mousa, Layan A Al Abbas, Noor M Al Zayer, Muhanned M Alqhtani, Yahya Naguib

**Affiliations:** 1 Surgery, Arabian Gulf University, Manama, BHR; 2 Physiology, Arabian Gulf University, Manama, BHR

**Keywords:** cesarean section, magnetic resonance imaging, abdominal wall mass, surgical scar, endometriosis

## Abstract

Endometriosis is the presence of ectopic functioning endometrial tissue outside the uterine cavity. Scar endometriosis is a rare condition that typically follows obstetrical and gynecological surgeries. Although the symptoms are non-specific and varying, scar endometriosis classically presents with cyclic pain at the site of incision during menstruation. The diagnosis of scar endometriosis remains challenging and requires a comprehensive approach, including clinical presentation and histological and radiological findings. Here, we present a case of extragonadal endometriosis at the cesarean section scar. The patient presented with cyclical menstrual pain at the surgical incision. Our aim in this case report is to present the approach to diagnosing such a condition with the associated presentation and histological findings.

## Introduction

Endometriosis is a gynecological disease where ectopic endometrial tissue is present outside the uterine cavity. It is affected by fluctuations in female sex hormones [1]. It commonly occurs anywhere in the abdominal and pelvic cavity, such as the ovaries, pelvis, pouch of Douglas, uterine ligaments, and the abdominal wall [2]. Other rare locations are the kidney, bladder, lungs, and brain [3]. Instances of abdominal wall endometriosis have recently increased due to increased cesarean section (C-section) scars where endometrial tissue implants at the scar location postoperatively. The prevalence of C-section scar endometriosis ranges between 0.2% and 0.8%. Classically, C-section scar endometriosis presents in women aged between 21 and 47 years with a mean age of 32 years [2,3].

## Case presentation

A 32-year-old female patient presented to our surgical clinic with a history of lower abdominal wall swelling, which increased in size during menstruation. The swelling was associated with cyclical pain during menstruation since she had a lower segment cesarean section (LSCS) performed two years earlier.

On physical examination, there was a tender, palpable 3 x 4 cm lump within the right lateral border of the previous LSCS scar.

**Figure 1:**
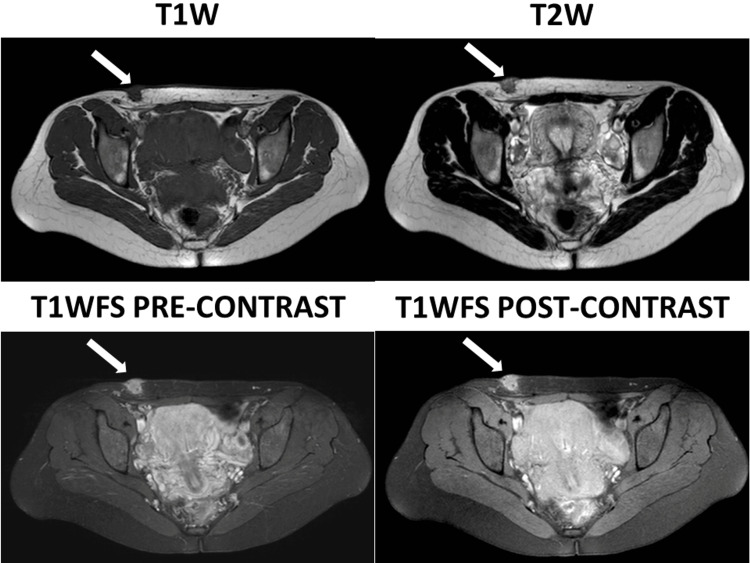
Magnetic resonance imaging of the pelvis showing an anterior abdominal wall lesion (white arrows), highly suggestive of endometrioma.

Pelvic magnetic resonance imaging (MRI) revealed a 13 mm rounded, well-defined hypointense lesion in the T1-weighted sequence, showing the intermediated signal in the T2-weighted sequence at the dermis, epidermis, and subcutaneous tissue of the right lower anterior abdominal wall. It showed mild homogenous enhancement, highly suggestive of endometrioma (Figure 1).

An excisional biopsy with free margin was performed, and the specimen was sent for histopathological interpretation.

On gross examination, abdominal wall (skin/soft tissue) mass excisional biopsy sectioning revealed a relatively well-demarcated solid tarnished white subcutaneous nodule with punctuate hemorrhage and microcyst changes. This nodule measured 22 mm (medial/lateral) × 17 mm (anterior/posterior) × 15 mm (superior/inferior). It was excised entirely (>10 mm) but was 8 mm away from the medial margins.

Microscopically, the mass of the abdominal wall (skin/soft tissue) suggested endometriosis. The classical ectopic endometrial glands/stroma formed the lesion and was associated with variable degrees of hemorrhage and fibroblastic scaring tissue reaction. No atypia was seen. The lesion was excised entirely at all surgical excision margins.

A diagnosis of C-section scar endometriosis was reached. The patient recovered well postoperatively, with resolved cyclical pain and associated swelling. A two-year follow-up showed no recurrence.

## Discussion

Endometriosis is defined by the presence of endometrial glands and stroma outside the uterus [4]. Although the pathogenesis of endometriosis remains unclear, Sampson’s theory of retrograde menstruation is the most accepted hypothesis. However, other factors must be at play in the development of endometriosis lesions, as this mechanism is also present in healthy women. This involves a complex interplay of congenital, environmental, epigenetic, autoimmune, and allergic factors [3].

According to a systematic review and meta-analysis, the prevalence of endometriosis in Middle Eastern women who had laparoscopy was approximately 12.9% [5]. Scar endometriosis can be seen after procedures such as C-sections, episiotomy, hysterectomy, and tubal ligation [6]. In addition, the incidence of endometriosis after cesarean section ranges between 0.03% and 0.4%. There is a significant increase in the incidence of scar endometriosis associated with the increased C-section rate [5,7].

Endometriosis can present with various degrees of symptoms or no symptoms at all. An example of symptoms at presentation can be a painful mass that may be accompanied by dysmenorrhea, palpable abdominal mass, and growing mass during the menstrual cycle located at or near the C-section incision scar [2].

Imaging plays a crucial role in the diagnosis and management of endometriosis. Moreover, ultrasonography helps estimate the size of the endometriosis nodule and determine if it is infiltrating the abdominal fascia [8]. During the evaluation of the mass, ultrasonography, computed tomography, and MRI help provide information about the location of the mass as well as its size and volume [9].

Although many imaging modalities are used, MRI is considered the superior imaging modality that aids in characterizing an endometriotic lesion, ruling out other differential diagnoses and guiding preoperative planning [10]. Our patient presented with a painful growing mass during the menstrual cycle, and MRI was the modality used to investigate the lesion.

When a swelling occurs at the surgical site incision, a differential diagnosis is established, such as hernia, hematoma, keloid, adenomyosis, leiomyoma, fibroma, lipoma, abdominal adhesions, peritoneal carcinomatosis or desmoid tumors. A detailed history, physical examination, and the use of imaging help to narrow differentials. However, histological findings play a significant role in confirming the diagnosis [11]. Although C-section scar endometriosis is a rare form of endometriosis, our case was a typical clinical radiological presentation.

The mainstay of treatment and diagnosis of C-section scar endometrioses is surgical resection with free margin as medical treatment only has a high failure rate. To prevent extrauterine LSCS endometriosis, thorough cleaning is recommended during the closure of the LSCS, especially both the corner sites of the adipose layer and the fascia layer [1,12].

## Conclusions

We presented a rare case of an LSCS scar endometriosis with typical cyclic pain at the surgical scar at the time of menstruation. We provided a comprehensive approach to diagnosing such a rare condition. Diagnosis of extragonadal endometriosis must embrace the associated presentation, as well as radiological and histological findings. Recurrence of C-section endometriosis can be prevented by proper diagnostic and surgical strategies.
